# Microencapsulation of Phenolic Extracts from *Verbascum sinaiticum* Leaf Using Maltodextrin and Gum Arabic: Physicochemical Properties, Encapsulation Efficiency, and Storage Stability

**DOI:** 10.3390/molecules31030471

**Published:** 2026-01-29

**Authors:** Alemu Belay Legesse, Shimelis Admassu Emire, Timilehin Martins Oyinloye, Won Byong Yoon

**Affiliations:** 1 Department of Food Science and Biotechnology, College of Agriculture and Life Sciences, Kangwon National University, Chuncheon 24341, Republic of Korea; alemubelay@dbu.edu.et (A.B.L.); oyinloyetm@kangwon.ac.kr (T.M.O.); 2 School of Chemical and Bioengineering, Institute of Technology, Addis Ababa University, Addis Ababa P.O. Box 385, Ethiopia; shimelis.admassu@aait.edu.et; 3 Department of Food Engineering, College of Engineering, Debre Berhan University, Debre Berhan P.O. Box 445, Ethiopia; 4 Department of Food Biotechnology and Environmental Science, Kangwon National University, Chuncheon 24341, Republic of Korea; 5 Elder-Friendly Food Research Center, Agriculture and Life Science Research Institute, Kangwon National University, Chuncheon 24341, Republic of Korea

**Keywords:** encapsulation, bioactive compound, in vitro digestion, freeze dryer, phenolic compounds, XRD, SEM

## Abstract

*Verbascum sinaiticum* (*V. sinaiticum*), locally known as “Qetetina” in Ethiopia, is a rich source of phenolic compounds, but its stability and sensory limitations restrict its application. This study evaluated the physicochemical, functional, morphological, structural, storage stability, and in vitro digestibility characteristics of *V. sinaiticum* extract encapsulated with 100% Gum Arabic (GA), 100% maltodextrin (MD), and a mixed coating material (MD:GA = 8:2 ratio) using freeze-drying. MD-based microcapsules demonstrated superior functional properties, including high water solubility (98.48%), low moisture content (1.36%), low hygroscopicity (14.61%), and high encapsulation efficiency (81.31%). During 32 days of storage, MD microcapsules retained 71.84% of total phenolic content and showed minimal release under gastric and thermal conditions, while GA encapsulates were less stable. Structural analysis using XRD, FTIR, and SEM confirmed successful encapsulation, with compact morphology, consistent phenolic preservation, and structural integrity. Furthermore, encapsulation masked the undesirable sensory attributes of the extract, enhancing its potential use in nutraceuticals. Overall, freeze-drying with MD provided the most effective encapsulation system, significantly improving the stability and storage potential of *V. sinaiticum* extract.

## 1. Introduction

*Verbascum sinaiticum* (*V. sinaiticum*), locally known as “Qetetina” in Ethiopia, is a rich source of phenolic compounds, secondary metabolites with significant application in pharmaceutical, chemical, and food industries [1,2,3,4,5]. These phenolic compounds, including flavonolignans, phenylethanoid glycosides (e.g., verbascoside), and flavonoids (e.g., quercetin and rutin), exhibit strong antioxidant, antimicrobial, and anti-inflammatory activities, supporting their role in mitigating oxidative stress, inhibiting foodborne pathogens, and offering therapeutic potential [5,6,7,8]. Optimized extraction techniques, such as freeze-drying, ethanol extraction, and ultrasound-assisted methods, have yielded high total phenolic content (up to 181.73 mg GAE/g) and enhanced bioactivity from *V. sinaiticum* leaves [3,4].

Despite their remarkable bioactivity, phenolic compounds are highly unstable due to their unsaturated structures, which are prone to degradation under heat, light, oxygen, and storage conditions [1,9,10]. In addition, their bitter and astringent taste often reduces consumer acceptability in food formulations [1,11]. These dual challenges, i.e., instability and adverse sensory properties, necessitate protective delivery systems that preserve phenolic compounds while improving their usability.

Microencapsulation has emerged as a promising strategy to address these challenges. By entrapping bioactives within a carrier matrix, microencapsulation shields them from environmental stress, improves solubility and bioavailability, masks undesirable flavors, extends shelf life, and enables controlled release during digestion [1,9,12,13]. The choice of wall material is critical, as it determines encapsulation efficiency, stability, and release behavior. Polysaccharides such as maltodextrin (MD), Gum Arabic (GA), chitosan, and modified starches are widely used for food applications. MD offers low viscosity, high solubility, and strong film-forming ability [11], while GA is valued for emulsification and oxidative stability [14]. When used in combination, MD and GA provide synergistic effects, achieving encapsulation efficiencies ranging from 50.9% to 98.01% depending on formulation [15,16,17]. These blends have successfully preserved phenolics in extracts from sources such as *C. caudatus*, *Moringa oleifera*, hop, and tomato juice, maintaining antioxidant activity and stability during storage [18,19,20,21].

Studies further highlight that GA–MD systems enhance both thermal and oxidative stability during freeze-drying and storage, reduce compound leakage under humid conditions, and enable targeted release under gastrointestinal conditions, with greater release observed in intestinal (pH 7.4) than gastric (pH 2.2) environments [9,20,21,22,23]. Such properties make GA–MD blends particularly relevant for functional food and nutraceutical applications, where stability, bioavailability, and sensory quality are essential [17,24]. In contrast to pharmaceutical applications that often rely on synthetic polymers (e.g., PLGA, chitosan), food systems favor natural polysaccharides like GA and MD due to their safety and regulatory acceptance [25,26].

Despite extensive research on microencapsulation of plant-derived phenolics, the application of GA, MD, and their blends to encapsulate *V. sinaiticum* extracts remains unexplored. To address this gap, the present study employed freeze-dried ethanol extracts of *V. sinaiticum* leaves and evaluated their encapsulation using GA, MD, and their blend (MDGA = 8:2 *w*/*w*). Specifically, the study aimed to (1) assess the efficacy and physical properties of encapsulated *V. sinaiticum* extracts, (2) characterize their morphology and structural integrity through FTIR, SEM, and XRD analyses, and (3) determine their storage stability at 4 °C, 25 °C, and 45 °C over 32 days. This is the first report on encapsulating *V. sinaiticum* extracts using MD, GA, and their blends, with findings expected to provide insights into industrial applications for enhancing the stability, sensory quality, and shelf life of plant-based bioactives in food and pharmaceutical systems.

## 2. Materials and Methods

### 2.1. Materials

The leaf powder of *V. sinaiticum* was prepared using the methodology outlined in Legesse et al. [3]. Maltodextrin (Dextrose Equivalent (DE) 16.5–19.5) and gum arabic (acacia powder) were purchased from Sigma Aldrich Merck, Seoul, Republic of Korea. All reagents and chemicals used were of analytical grade, including methanol, ethanol, HPLC-grade water, sodium nitrite, sodium hydroxide, Folin–Ciocalteu reagent, sodium carbonate, potassium persulfate, catechin, gallic acid, aluminum chloride, sodium chloride, hydrochloric acid, pancreatin, pepsin, monobasic potassium phosphate, maltodextrin, Gum Arabic, and simulated gastric fluid, all sourced from Sigma Aldrich Merck, Seoul, Republic of Korea.

### 2.2. Extraction Procedure

The maceration extraction (ME) of *V. sinaiticum* leaves was performed following an adapted protocol from Mahdavi et al. [27]. Fresh leaves were cleaned, washed, and freeze-dried for 72 h before being ground (Dietz-Motoren GmbH & Co. KG, Dettingen unter Teck, Germany) and sieved (20-mesh). The resulting powder (3 g) was mixed with 90 mL of 70% ethanol (1:30 *w*/*v* ratio) and extracted at 30 °C for 24 h in a shaking water bath (JSSB-50T, JS Research Inc., Gongju-city, Republic of Korea). The supernatant was collected and stored at −80 °C for subsequent analysis.

Following maceration, the extract slurry was separated from the solid plant residue by vacuum filtration using Whatman No. 1 filter paper, ensuring complete removal of insoluble materials prior to further processing. The resulting filtrate was then concentrated under reduced pressure using a rotary evaporator (Büchi, Flawil, Switzerland) at 40 °C with a rotation speed of 120 rpm, until complete removal of the extraction solvent (ethanol). The concentrated extract was subsequently adjusted to a final solid content of approximately 10% (*w*/*w*) to obtain a standardized extract concentration. The extract was then aliquoted and stored at −80 °C until further analyses and microencapsulation experiments. These processing conditions were carefully selected to minimize thermal and oxidative degradation of phenolic compounds while ensuring adequate extract concentration and stability. The extract yield (%) was computed as:

(1) Extract yield(%)=(Extract weight(We)Intital sample weight(Wd))×100

where W_e_ = weight of the dried extract after solvent evaporation (g); W_d_ = initial dry weight of the plant material used for extraction (g).

### 2.3. Preparation of Microencapsulation Mixture 

The optimal wall material formulation for encapsulating *V. sinaiticum* extract was determined based on preliminary trials evaluating range of MD to GA ratios (*w*/*w*): (0:10, 1:9, 2:8, 3:7, 4:6, 7:3, 8:2, 9:1, 10:0, 0:20, and 20:0) (Supplementary Material Table S1). Among these, the MDGA ratio of 8:2 (*w*/*w*) was selected as the most suitable combination, providing the best compromise between emulsification capacity, structural integrity, and encapsulation efficiency. This selection was guided by observed improvements in emulsion stability, encapsulation morphology, and preliminary encapsulation performance. A 10% MD solution (10 g MD/90 g DW) was prepared by dissolution at 27 °C with agitation (70 rpm, 12 h), while a 4% GA solution for 1 h (4 g GA/96 g DW) was freshly prepared two hours before use [28]. These were blended using a magnetic stirrer (Heidolph MR 3001K, 1250 rpm, Schwabach, Germany) to achieve the target ratio and 10% total solids content (Table 1).

For phenolic extraction, dried *V. sinaiticum* leaves were extracted, centrifuged (4000 rpm, 15 min), and concentrated via rotary evaporation (40 °C, 100 mbar). The 70% ethanol concentrate was frozen (−80 °C, 72 h) and lyophilized (72 h, −50 °C, 0.1 mbar) to produce a stable phenolic powder stored in light-protected containers with desiccant.

Encapsulation was performed by homogenizing the coating solution with the freeze-dried *V. sinaiticum* extract at a 10:1 coating-to-core (*V. sinaiticum* extract) ratio using an Ultra-Turrax T25 homogenizer at 12,000 rpm (IKA-Werke GmbH & Co. KG, Staufen im Breisgau, Germany). The 10:1 ratio was selected based on previous studies [29] and preliminary trials evaluating various coating-to-core ratios (1:1, 5:5, 1:5, 10:1, 1:10, 20:1, and 1:20), which indicated superior encapsulation efficiency and structural integrity at this proportion. The mixture was frozen (−80 °C, 2 h), lyophilized (72 h), then ground and sieved (20-mesh). All preparations were performed in Triplicate.

### 2.4. Freeze Drying of Encapsulation Conditions

Following Saikia et al. [28], the combined solutions were frozen at −80 °C for 2 h (CLN-51U, Nihon Freezer, Tokyo, Japan) before freeze drying at −55.8 °C and 0.40 mbar for 72 h (FD 8508, IIShinBioBase, Daejeon, Republic of Korea). The resulting bioactive matrix was ground, sieved (20-mesh), and stored in sealed aluminum containers at 4 °C for analysis.

### 2.5. Moisture Content (MC) and Water Activity (aw)

The MC of Freeze-dried 70% ethanol crude *V. sinaiticum* leaf extract was determined by gravimetric analysis. Samples were weighed and oven-dried at 105 °C with an air circulation speed of 1.5 m/s for 24 h [30]. The water activity (aw) of the freeze-dry encapsulates was measured using an Aqualab TDL (aw) (Meter Group Inc., Pullman, WA, USA), with all measurements conducted in triplicate [30].

### 2.6. Hygroscopicity

Hygroscopicity was measured to determine the moisture absorbed by *V. sinaiticum* encapsulated freeze-dried powder from the storage environment. The method was adapted from [30]. 1 g of each sample was placed in Petri dishes and stored in sealed containers maintained at 25 °C and 75% relative humidity, regulated with a saturated sodium chloride (NaCl) solution. After 15 days of incubation, the samples were reweighed, and hygroscopicity was determined using Equation (1):

(2) Hygroscopicity=(W2−W1W1)×100

where W_1_ is the initial sample weight and W_2_ is the sample weight that remains following the hygroscopicity treatment.

### 2.7. Bulk and Tapped Density

Bulk and tapped densities were determined following the protocol by Dadi et al. [31]. 5 g of encapsulated freeze-dried powders were placed in a 25 mL measuring cylinder, and the occupied volume was recorded to calculate bulk density using Equation (3). For tapped density, the cylinder was gently tapped on a laboratory benchtop until a constant volume was achieved. Bulk density (ρ_e_) was calculated as:

(3) $$Bulk\;Density\left(\frac{g}{mL}\right)=\frac{weight\;of\;microcapsule}{occupied\;volume}$$

### 2.8. Flowability

Flow properties of the encapsulated freeze-dried powder were assessed based on the method by George et al. [9] with minor modifications. Measurements included the angle of repose, Hausner’s ratio, and Carr’s index. The angle of repose was measured using a powder flow tester (AMETEK Brookfield PFT, Middleboro, MA, USA) as described in Equations (4)–(6):

(4) $$Hausner^{\prime }s\;ratio=\frac{Tapped\;density}{Bulk\;density}$$

(5) $$Carr^{\prime }s\;index=\frac{\left(Tapped\;density-Bulk\;density\right)}{Tapped\;density}\times 100$$

(6) $$Angle\;of\;repose\left({}^{\circ}\right)={tan}^{-1}\left(\frac{H}{R}\right)$$

where R is the radius at the base and H is the height of the pile.

### 2.9. Water Absorption Index (WAI) and Water Solubility Capacity (WSC)

The Water Absorption Index (WAI) of the 1 g of the *V. sinaiticum* encapsulated freeze-dried powder bioactive product was determined with a slight modification to the method by George et al. [9]. 1 g of encapsulated freeze-dried powder was weighed into a pre-weighed 50 mL centrifuge tube, mixed with 12 mL of distilled water, and shaken in a water bath at 30 °C for 30 min. The mixture was centrifuged at 1915× *g* for 15 min, and the supernatant was decanted. The remaining hydrated gel was weighed, and WAI was calculated using Equation (7):

(7) $$Water\;absorption\;index\left(WAI\right)=\frac{weight\;of\;hydrated\;gel}{weight\;of\;an\;initial\;sample}$$

For Water Solubility Capacity (WSC), the collected supernatant was dried overnight at 105 °C in a pre-weighed aluminum container. WSC was calculated using Equation (8):

(8) $$Water\;solubility\;capacity\left(WSC\right)=\frac{weight\;of\;dissolved\;solids}{weight\;of\;an\;initial\;sample}$$

### 2.10. Determination of Total Polyphenol Content (TPC)

Total phenolic content (TPC) was determined using the Folin–Ciocalteu method, following the procedure described in our previous study [3]. During the procedure, 0.2 mL of extract was mixed with 2.5 mL of 10% Folin–Ciocalteu reagent, followed by the addition of 2 mL of 7.5% sodium carbonate solution (75 g/mL). The mixture was thoroughly combined and incubated in the dark at room temperature for 2 h. The same procedure was applied to blanks and gallic acid standards at various concentrations to generate a standard curve. Absorbance was measured at 765 nm using a Spectra i3x plate reader (Molecular Devices, LLC, Seoul, Republic of Korea).

### 2.11. Microencapsulation Efficiency (MEE)

George et al. [9] provided a modified version of the approach used to determine the encapsulation efficiency. To ascertain the TPC, 100 mg of the *V. sinaiticum* sample product was dissolved in 1 mL of distilled water with agitation. After adding 9 mL of ethanol, the liquid was stirred for 5 min. After that, the mixture was passed through a membrane filter with 0.45 μm-sized pores. To extract surface phenolic compounds from the microcapsule wall, an additional 100 mg of the microencapsulated product was mixed with 10 mL of ethanol, vortexed for 10 s, then centrifuged at 1915× *g* for 3 min. The translucent supernatant was collected and filtered using a 0.45 μm membrane filter. Finally, the total and surface phenolic compounds were quantified as indicated in the subsequent section. Equation (9) was utilized to ascertain the MEE.

(9) $$MEE\left(\%\right)=\frac{\left(TPC-SPC\right)}{TPC}\times 100$$

where MEE stands for microencapsulation efficiency, TPC: total phenolic content within the core of the encapsulate, and SPC: surface phenolic content.

The SPC is expressed as the number of phenolic compounds extracted from the surface, normalized to the total weight of the encapsulated particles. The formula used is:

(10) SPC(mg GAEg sample)=C×VW

where C: Concentration of phenolic compounds in the extract (mg GAE/mL), often determined using a standard curve (GAE: gallic acid equivalence), V: Volume of solvent used for extracting surface phenolic compounds (mL) and W: Weight of the encapsulated particles (g).

### 2.12. Storability

Encapsulated freeze-dried powder and 70% ethanol leaf extract (without encapsulants) were stored at 4 °C, 25 °C, and 45 °C for 32 days to assess storability with minor modification [9,20]. Total phenolic content (TPC) was measured weekly using the Folin–Ciocalteu method with six experimental replicates. After 32 days, the percentage loss of TPC was calculated.

### 2.13. In Vitro Digestibility

#### 2.13.1. Simulated Gastric Fluid (SGF)

Simulated gastric fluid (SGF) was prepared according to Saikia et al. [28] with minor modifications. Briefly, 3.2 g of pepsin from porcine gastric mucosa (≥2500 U/mg protein; Sigma-Aldrich, St. Louis, MO, USA) and 2.0 g of sodium chloride were dissolved in 7 mL of hydrochloric acid and diluted to 1000 mL with distilled water. The pH was adjusted to 2.0 ± 0.05 using 1.0 M hydrochloric acid and verified with a calibrated digital pH meter (three-point calibration at pH 1.68, 4.01, and 7.00). For the gastric digestion assay, 0.2 g of microencapsulated sample was mixed with 2.4 mL of SGF in a 40 mL centrifuge tube and incubated at 37 ± 0.5 °C with continuous shaking at 80 rpm for 2 h. After incubation, the samples were centrifuged at 1915× *g* and 4 °C for 10 min. The supernatant was filtered and neutralized to pH 7.0 ± 0.05 using 1.0 M NaOH prior to total phenolic content (TPC) determination using the Folin–Ciocalteu method as previously described [3]. All experiments were conducted in triplicate.

#### 2.13.2. Simulated Intestinal Fluid (SIF)

SIF was prepared following [28]. Initially, 6.8 g of monobasic potassium phosphate was dissolved in 250 mL of water. The volume was adjusted to 1000 mL, and the pH was set to 6.8. Then, 10 g of pancreatin and 77 mL of 0.2 N sodium hydroxide were added. A 40 mL tube containing 0.2 g of microencapsulated product and 4.8 mL of simulated intestinal fluid (SIF) was incubated at 37 ± 0.5 °C for 2 h (static conditions). The mixture was then cooled, centrifuged (1915× *g*, 4 °C, 10 min), and filtered (0.45 μm). For analysis, the supernatant was acidified with 0.2 N HCl to terminate enzymatic activity. The supernatant was filtered and neutralized before TPC determination using the previously described Folin–Ciocalteu method [3].

### 2.14. Structural Characterization

Scanning Electron Microscopy (SEM) and X-ray diffraction (XRD) were employed to assess the crystallization phases and morphology of the 70% ethanol leaf extract and the encapsulated freeze-dried powder, respectively. For XRD analysis, monochromatic light with a wavelength of 0.154 nm was generated using a source operating at 40 kV and 40 mA (XRD6000, Shimadzu, Kyoto, Japan). The samples were scanned at room temperature over a 2θ range of 5–40° at 0.04° intervals, with a scanning speed of 2° per minute [31]. For SEM (Thermo Scientific Phenom Desktop, Waltham, MA, USA) analysis, the encapsulated freeze-dried powder was coated with a thin Au-Pd layer and analyzed at 5 kV with magnifications up to 10,000× [3].

### 2.15. Attenuated Total Reflection-Fourier Transform Infrared Spectroscopy (ATR-FTIR)

The *V. sinaiticum* was placed on the diamond crystal surface of the attenuated total reflection (ATR) cell of the FTIR spectrometer (model: iS50, Thermo Scientific, Waltham, MA, USA). The FTIR analysis covered wave numbers from 400 cm^−1^ to 4000 cm^−1^, with an average scanning rate of 1 cm^−1^ resolution [3].

### 2.16. Statistical Analysis

JMP Pro Version 17 statistical analysis was conducted using one-way analysis of variance (ANOVA) followed by Tukey’s Post Hoc test to determine significant differences among encapsulated samples at a 95% confidence level (*p* < 0.05). Results are reported as mean ± standard deviation.

## 3. Results and Discussions

### 3.1. Physical Properties of Encapsulated V. sinaiticum Product

The physical properties of the encapsulated *V. sinaiticum* product, included moisture content, hygroscopicity, water activity, water absorption capacity (WAC), water solubility index (WSI), bulk and tapped density, and flowability were comprehensively evaluated (Table 2).

#### 3.1.1. Water Activity (aw) and Moisture Content (MC)

Water activity (aw) and moisture content (MC) are a critical factor influencing the stickiness, flowability, stability and storability of microcapsules [9]. The MC ranged from 1.36% for MD to 1.66% for GA (Table 2), and both MC and aw were significantly affected by the coating materials (*p* < 0.05). These values are within the range previously reported for similar encapsulation systems, including phenolic compounds and plant extracts, using MD, GA, or their combinations [9,28,29,31,32,33]. Table 2 shows that the aw values of the freeze-dried encapsulates were consistently low (≤0.3), indicating minimal microbial and chemical degradation risk. This aligns with findings by Todorović et al. [32], who highlighted that reduced aw enhances phenolic stability by limiting molecular mobility and slowing degradation. Overall, the low MC and aw observed here suggest strong resistance to deterioration and good suitability for industrial applications, minimizing storage problems such as caking and ensuring stable functionality in dry formulations.

#### 3.1.2. Hygroscopicity

The encapsulated freeze-dried powder bioactive compounds exhibited hygroscopicity levels ranging from 10.57% to 14.61%, with MDGA (10.57 ± 0.42%) showing significantly lower values (*p* < 0.05) than MD (14.61 ± 0.04%) and GA (13.38 ± 0.31%). This indicates that MDGA microcapsules possess superior moisture resistance, which is advantageous for storage stability in humid environments (Table 2). These findings align with previous studies [9,31]. MD-encapsulated freeze-dried powder exhibited the highest hygroscopicity, likely due to its high moisture absorption capacity associated with hydroxyl functional groups [34,35]. Among the microcapsules evaluated, MDGA formulations demonstrated the lowest hygroscopicity, suggesting possible chemical interactions between the *V. sinaiticum* extract and the mixed coating materials, leading to enhanced moisture resistance. A significant difference (*p* < 0.05) was observed between the hygroscopicity of MDGA and both MD and GA microcapsules. The reduced hygroscopicity of MDGA microcapsules enhances their storability and minimizes moisture-induced deterioration, making them preferable for prolonged storage compared to MD and GA microcapsules [9]. This reduced hygroscopicity of MDGA is particularly crucial for maintaining product quality in diverse climatic conditions and for preserving the integrity of active compounds during extended shelf life, reducing the need for costly desiccant packaging in certain food matrix applications.

#### 3.1.3. Water Solubility Index (WSI) and Water Absorption Capacity (WAC)

According to George et al. [9], the solubility characteristics of microcapsules play a crucial role in their reconstitution. The rehydration behavior of microencapsulated powders is a critical quality attribute that governs their functional performance in aqueous food and nutraceutical systems. In this study, all microcapsule formulations exhibited high WSI values (86.51–98.48%; Table 2), confirming that freeze-drying combined with polysaccharide-based wall materials produces readily dispersible powders. However, the magnitude of solubility differed significantly among formulations (*p* < 0.05), underscoring the dominant role of wall material chemistry in controlling hydration dynamics.

Maltodextrin-based microcapsules demonstrated the most rapid and complete dissolution, reflected by the highest WSI (98.48%). This behavior is attributed to the relatively short glucose chains and high amorphous content of maltodextrin, which facilitate fast water diffusion and polymer disentanglement during reconstitution [36,37,38,39]. In contrast, Gum Arabic microcapsules showed lower WSI values (86.51%), likely due to their complex, highly branched macromolecular architecture and protein–polysaccharide associations, which slow matrix swelling and dissolution [27]. The intermediate WSI observed for the MDGA system indicates that blending the two polymers alters the hydration pathway by forming a more structured polymer network. In this case, hydrogen bonding and physical chain interpenetration between MD and GA partially limit water access without fully suppressing solubility [26,40]. From a formulation standpoint, these differences suggest that MD-based powders are better suited for applications requiring instantaneous dissolution, whereas GA-containing systems, particularly MDGA, may be advantageous where moderated hydration or gradual release of phenolic compounds is desirable.

Water absorption capacity (WAC) further differentiated the encapsulation systems. GA microcapsules exhibited the highest WAC (Table 2), reflecting the strong water-binding ability of high-molecular-weight polysaccharides rich in hydrophilic functional groups [41]. In contrast, the MDGA formulation showed the lowest WAC, indicating restricted water uptake. This reduction can be attributed to matrix densification arising from polymer–polymer interactions, which decreases the availability of free hydrophilic sites. From a technological perspective, low WAC is beneficial, as it reduces moisture sensitivity, improves powder flow, and limits caking during storage under humid conditions [42]. Collectively, the WSI–WAC relationship demonstrates that polymer blending offers a practical approach to fine-tuning hydration behavior without compromising solubility.

The intermediate water solubility index (WSI) and reduced water absorption capacity (WAC) observed for the MDGA microcapsules can be attributed to physicochemical interactions between maltodextrin and Gum Arabic, rather than to the formation of new covalent bonds. Maltodextrin is a low–molecular-weight polysaccharide with high water solubility, whereas Gum Arabic is a high-molecular-weight, branched polysaccharide containing numerous hydrophilic functional groups and proteinaceous fractions. When combined, MD and GA interact predominantly through hydrogen bonding between hydroxyl groups and physical entanglement of polymer chains, leading to the formation of a more compact and cohesive encapsulating matrix. This matrix densification reduces pore volume and limits water penetration and polymer swelling, resulting in lower WAC compared with GA-only microcapsules. Simultaneously, the presence of GA moderates the inherently high solubility of MD, explaining the intermediate WSI values observed for the MDGA formulation. Furthermore, partial occupation of hydrophilic sites through MD–GA interactions reduce the availability of free water-binding groups, contributing to lower hygroscopicity and improved moisture resistance. Similar mechanisms involving polymer–polymer hydrogen bonding, matrix densification, and reduced water accessibility have been widely reported for MDGA-based encapsulation systems used for plant extracts and bioactive powders [9,26,38].

#### 3.1.4. Bulk Density and Tapped Density

Powder density characteristics provide insight into particle packing behavior, storage efficiency, and susceptibility to oxidative degradation. In the present study, bulk density values varied from 0.20 to 0.35 g/mL (Table 2), with statistically significant differences among formulations (*p* < 0.05). MD and MDGA microcapsules exhibited higher bulk densities than GA microcapsules, indicating a more compact particle arrangement with reduced interparticle voids.

The lower bulk density observed for GA-coated microcapsules is likely associated with the high viscosity of GA solutions prior to freeze-drying, which promotes the formation of larger, more porous structures. Such porosity increases the volume occupied per unit mass, leading to reduced packing efficiency. In contrast, the relatively low viscosity and flexible chain structure of maltodextrin favor the formation of denser matrices, which is reflected in the higher bulk densities of MD-containing systems [26,27]. These differences have practical implications, as higher bulk density powders require less storage space and are less prone to oxidation due to reduced air entrapment.

Tapped density measurements followed a similar trend, with MD microcapsules showing the highest values and GA microcapsules the lowest. The MDGA system displayed intermediate tapped density, indicating that polymer blending influences particle rearrangement under mechanical stress. This behavior suggests that MD contributes to improved packing efficiency, while GA introduces structural heterogeneity that limits complete consolidation. Since tapped density directly affects handling, transportation, and filling operations, these results further highlight the importance of wall material selection when designing encapsulated powders for industrial-scale applications. Overall, the combined evaluation of hydration indices and density parameters demonstrates that maltodextrin, Gum Arabic, and their blend impart distinct yet complementary functional attributes. By adjusting wall material composition, it is possible to tailor solubility, moisture sensitivity, and packing behavior of phenolic-rich microcapsules to meet specific product and processing requirements.

#### 3.1.5. Flow Properties

Flow properties are crucial in food applications, influencing processing, packaging, and transportation. To comprehensively assess microcapsule flow behavior, angle of repose Hausner’s ratio, and Carr’s index were evaluated. The results, shown in Table 2, indicate that: Angle of repose ranged from 31.75° to 34.60°, Hausner’s ratio varied between 1.12 and 1.39, and Carr’s index ranged from 9.13% to 28.31%, according to Mahdavi et al. [27] and Chuyen et al. [39], poor flow properties are indicated by an angle of repose above 45°, a Carr’s index exceeding 25%, and a Hausner’s ratio above 1.25. In this study, MD and MDGA microcapsules showed moderate flowability, which can be attributed to their low moisture content and amorphous structural patterns, reducing cohesive forces and internal friction [9]. Significant differences (*p* < 0.05) were observed between MDGA and MD microcapsules in Carr’s index or Hausner’s ratio, indicating similar flow behavior. However, GA microcapsules exhibited poor flow properties, as indicated by their Hausner’s ratio (1.39) and Carr’s index (28.31%), despite having an angle of repose within the acceptable range. This suggests that GA microcapsules tend to form agglomerates due to their high viscosity, making them less suitable for applications requiring high flowability. Overall, MD and MDGA microcapsules demonstrated superior flow properties, making them better suited for food applications requiring ease of handling and mixing. The observed behavior is likely influenced by the lower viscosity of the coating materials and the relatively smaller surface area of MD and MDGA microcapsules. For food processing, the excellent flowability of MD and MDGA microcapsules is paramount. This property facilitates consistent dosing in automated filling machines, prevents blockages in processing lines, and ensures uniform mixing in powdered blends, thereby enhancing process efficiency and product quality.

The significant impact of coating materials on the solubility, density, water absorption, and flow properties of microcapsules. MD microcapsules exhibited the highest solubility and flowability, while GA microcapsules showed the lowest solubility and poorest flow properties. MDGA microcapsules, combining both MD and GA, exhibited intermediate solubility, density, and flow behavior, making them a balanced option for diverse food applications of *V. sinaiticum* especially in antimicrobial and antioxidant area. This is ideal for *V. sinaiticum* applications that need a balance of stability, controlled release, and easy processing—especially in functional bars or drinks where good dissolution and powder handling improve both performance and production.

The pure freeze-dried *V. sinaiticum* extract exhibited significantly higher hygroscopicity, moisture content, and water activity compared to encapsulated powders (*p* < 0.05), indicating lower physical stability. These findings confirm that wall materials effectively protect bioactive extracts and enhance powder functionality, handling, and storage stability.

### 3.2. Surface Morphology of Microcapsules of V. sinaiticum

**Figure 1:**
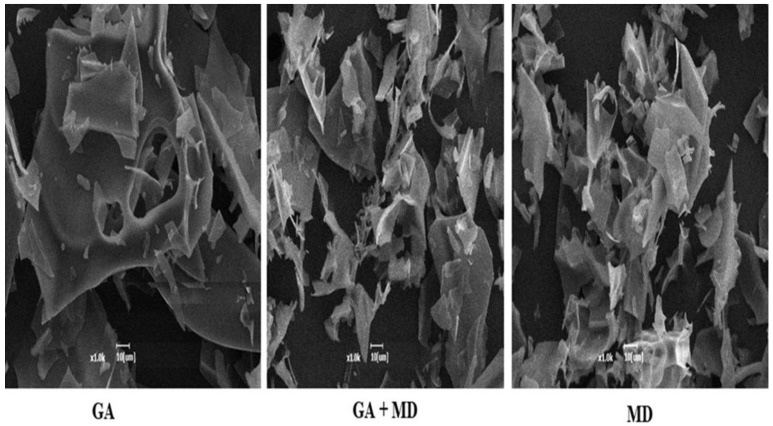
Morphological structures of microcapsules GA: Gum Arabic, and MD: maltodextrin-GA: mixed maltodextrin—gum Arabic.

Figure 1 presents SEM micrographs (×1000 magnification, scale bar = 10 µm) of freeze-dried *V. sinaiticum* extracts encapsulated with GA, MD, and their combination (MDGA, 8:2 *w*/*w*). Distinct morphological differences are evident among the samples, which reflect the influence of wall materials on structural integrity, active compound retention, and solubility.

GA-based microcapsules exhibit an irregular, porous, and brittle structure with visible cracks, likely due to rapid crystallization during freeze-drying. This porous nature enhances initial active compound release but may compromise long-term stability, consistent with findings by [40]. MD microcapsules are characterized by smaller, more fragmented flakes with a rough texture. The hydrophilic and low molecular weight nature of MD may enhance solubility but often results in poor film-forming and emulsifying properties, potentially compromising encapsulation stability. These observations align with Tonon et al. [35], who highlighted MD’s limitations as a sole encapsulation material.

In contrast, the MDGA microcapsules exhibit a more uniform, compact morphology with fewer cracks. This suggests improved mechanical stability and encapsulation efficiency, attributed to the complementary functional properties of MD and GA. The combination appears to mitigate the limitation of each individual coating material by enhancing both structural integrity and solubility.

### 3.3. FTIR Analysis of V. sinaiticum Microcapsules

**Figure 2:**
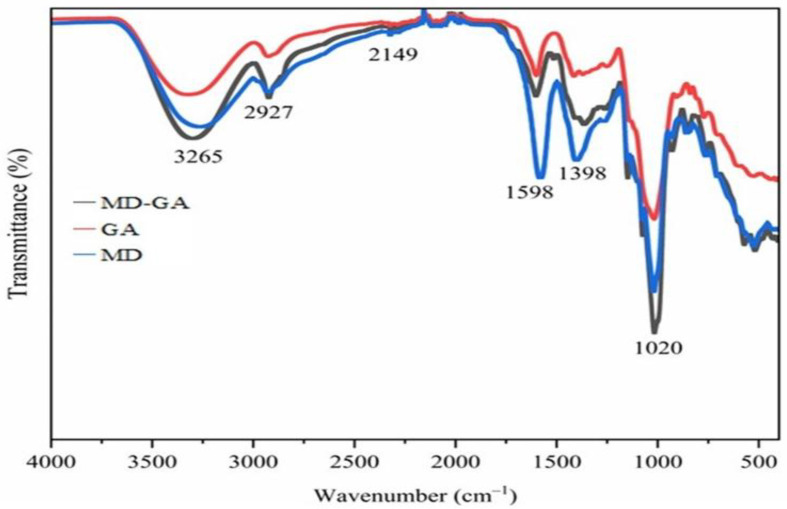
FTIR spectra of the obtained encapsulates, as denoted below individual lines: maltodextrin (MD) encapsulates, Gum Arabic (GA) encapsulates, and combination of MD-GA (8:2 *w*/*w*) encapsulates in freeze-dried extract.

FTIR spectroscopy (4000–400 cm^−1^) was used to evaluate functional group interactions and potential structural changes during encapsulation. Figure 2 shows the spectra of pure extract, GA, MD, and their mixture (MDGA = 8:2 *w*/*w*), allowing comparison of the core material with the wall matrices.

The spectrum of pure *V. sinaiticum* extract displayed characteristic phenolic peaks, including a broad O–H stretching band at 3265 cm^−1^, confirming the presence of hydroxyl groups typical of phenolics and carbohydrates [3]. Upon encapsulation, particularly in MDGA, this O–H band shifted slightly and broadened relative to the pure extract, indicating hydrogen bonding between phenolic groups and the wall materials. This FTIR spectral change, especially the broadening and displacement of the O–H stretching band, reflects strengthened intermolecular hydrogen bonding, which is directly associated with the modified hydration behavior of the microcapsules. A C–H stretching band at 2927 cm^−1^ corresponded to aliphatic chains present in both the wall polymers and the extract, while a weaker band near 2149 cm^−1^ was attributed to minor components of the coating materials.

In addition, the C=O and C–O stretching vibrations near 1020 cm^−1^, associated with polysaccharides and ester bonds, exhibited minor shifts in the MDGA system compared with MD or GA alone. These subtle changes, without the emergence of new absorption peaks, suggest weak non-covalent interactions, mainly hydrogen bonding and physical entrapment, rather than covalent modifications or degradation during freeze-drying. Such non-covalent interactions promote matrix densification and reduced availability of free hydrophilic sites, which explains the experimentally observed lower hygroscopicity and water absorption capacity (WAC) of the MDGA microcapsules, as well as their intermediate water solubility index (WSI). Thus, the general functional groups of the extract were preserved, confirming that encapsulation successfully stabilized the bioactive compounds.

Overall, the spectra of GA, MD, and MDGA retained their structural integrity, with MDGA showing the most favorable balance. The combination provided enhanced molecular interactions without compromising the chemical identity of either the extract or the wall matrices. The FTIR-derived structural evidence therefore supports the physicochemical performance of the MDGA system, linking molecular-level interactions to improved stability, moderated hydration behavior, and enhanced handling properties. This stability is essential for protecting sensitive compounds from environmental stressors, ensuring prolonged retention of bioactivity. While GA alone offered high encapsulation efficiency but weaker structural stability, and MD promoted solubility with lower cohesiveness, MDGA provided both improved solubility and stronger entrapment efficiency. These results reinforce previous findings that combining wall materials can optimize encapsulation outcomes [41]. The MDGA system, by leveraging the complementary properties of MD and GA, represents a promising strategy for applications in food and pharmaceuticals where both protection and controlled release of bioactive compounds are required.

### 3.4. The Crystallinity of Microcapsules Using X-Ray Diffraction (XRD)

**Figure 3:**
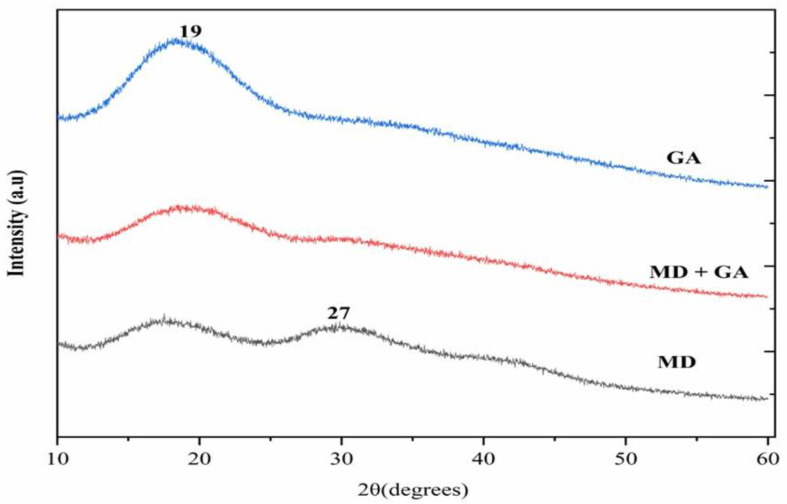
X-ray diffractogram of *V. sinaiticum* microcapsules in MD + GA, GA, and MD coatings. GA: gum arabic, and MD: maltodetrine, MD-GA: mixed maltodextrin—Gum Arabic.

Figure 3 illustrates the XRD patterns of GA, MD, and a MDGA mixture microcapsules and *V. sinaiticum* core extract [3]. All samples exhibited broad peaks with minimal crystallinity. The crystallinity of the microcapsules may influence their stability, as noted by [36,37]. Each encapsulated material displayed a broad peak centered at approximately 2θ = 19°, which is characteristic of amorphous polysaccharide matrices. In the MDGA microcapsules, the appearance of a weak additional feature at 2θ ≈ 27° does not indicate the formation of a crystalline phase, but rather reflects localized short-range molecular ordering arising from intermolecular interactions between maltodextrin and Gum Arabic. Similar amorphous diffraction behavior with secondary shoulders has been reported for blended polysaccharide systems used in encapsulation. Each encapsulated material displayed a broad peak at approximately 2θ = 19°, with a new signal at 2θ = 27° for MDGA, indicating an amorphous form, consistent with previous studies [1].

Specifically, the GA microcapsules showed a single broad peak centered around 2θ = 19°, consistent with its highly amorphous polysaccharide structure. In contrast, MD microcapsules showed two broads peaks at 2θ = 19° and 2θ = 27° peak, suggesting minor differences in their polymeric arrangement.

The presence of the weak 27° feature in MDGA microcapsules is associated with enhanced matrix compactness and reduced molecular mobility, which correlates with the experimentally observed lower hygroscopicity, moderated water absorption capacity, and improved stability of the MDGA formulation. These structural characteristics support the improved physicochemical performance of the MDGA system by limiting moisture penetration while maintaining sufficient amorphous character for effective solubility and release.

### 3.5. Microencapsulation on Efficiency (MEE), TPC, and SPC

Table 3 presents the encapsulation efficiency (MEE), surface phenolic content (SPC), and total phenolic content (TPC) for the freeze-dried powders. The initial TPC of the unencapsulated *V. sinaiticum* extract was 160.01± 0.78 mg GAE/g, which served as the baseline for evaluating encapsulating efficiency. The results indicate that freeze-drying increased both SPC and TPC values with higher MD content.

The encapsulation effectiveness of phenolic compounds from *V. sinaiticum* was evaluated based on the percentage of phenolic compounds retained within the matrix relative to the initial extract values. The findings show that the encapsulating material played a significant role in preserving phenolic compounds, with the highest retention observed using MD, the MDGA mixture, and GA, in that order. Encapsulation efficiencies ranged from 65% to 81.31%. In comparison, ref. [14] reported encapsulation efficiencies of 39–75% for MD-encapsulated cactus pear juice, and [28] found 78–97% encapsulation efficiencies for MD-encapsulated Averrhoa carambola pomace.

As shown in Table 3, MD encapsulation resulted in the highest TPC (107.00 ± 0.82 mg GAE/g) and SPC (20.00 ± 0.08 mg GAE/g), while GA showed the lowest TPC (40.07 ± 0.74 mg GAE/g) and SPC (11.04 ± 0.02 mg GAE/g). The MDGA combination yielded intermediate TPC (70.03± 1.51 mg GAE/g) and SPC (19.31±0.09 mg GAE/g). MD’s superior encapsulation capability, evidenced by higher TPC and SPC compared to GA, and the correlation between increased MD concentration in blends and higher SPC, is attributed to its robust film-forming and stabilizing properties. This trend was also supported by [40], who observed that freeze-dried encapsulates remained stable over extended storage periods. MD’s film-forming and stabilizing properties enhance encapsulation by creating a thicker, more uniform coating, leading to improved TPC retention and reduced SPC. This protective matrix minimizes phenolic degradation, thereby enhancing encapsulation efficiency and safeguarding bioactive compounds.

### 3.6. In Vitro Analysis Under Simulated Stomach and Intestinal Conditions

**Figure 4:**
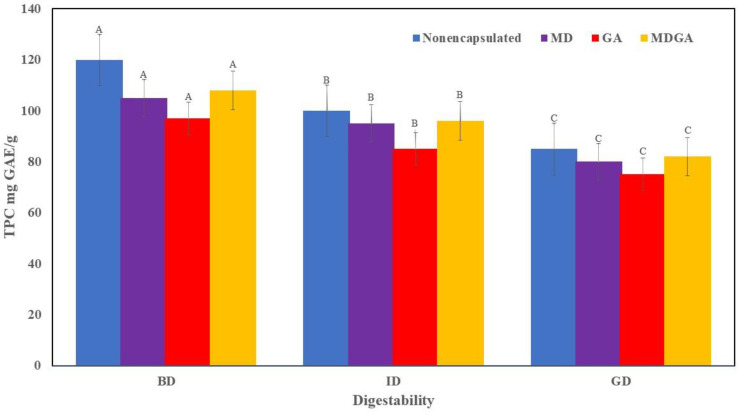
Digestibility of the microencapsulated bioactive products from *V. sinaiticum* leaves extract in terms of total phenolic content (TPC). Where GD = simulated gastric fluid, ID = simulated intestinal fluid, MD: maltodextrin, MDGA: maltodextrin and Gum Arabic mixture, freeze-dryer. Letters on the graph’s capital letters on BD: before digestibility, ID: digestion on intestine and on GD: digestion at gastric, shows insignificant differences at *p* > 0.05.

Figure 4 illustrates the in vitro gastrointestinal digestion behavior of both the non-encapsulated (pure) *Verbascum sinaiticum (V. sinaiticum*) extract and the microencapsulated formulations. Before digestion (BD), the non-encapsulated extract exhibited the highest total phenolic content (TPC), reflecting the absence of a protective matrix. However, during simulated gastric (GD) and intestinal digestion (ID), the pure extract showed a pronounced reduction in TPC, indicating its high susceptibility to acidic pH and digestive enzymes. In contrast, the microencapsulated systems enabled controlled phenolic release and enhanced protection against oxidative degradation of biomolecules such as lipids and DNA in the gastrointestinal tract [43].

Compared with the pure extract, all microencapsulated formulations (MD, GA, and MDGA) demonstrated significantly higher phenolic retention throughout digestion, confirming the protective role of the encapsulation matrix. The release of phenolic compounds from freeze-dried microcapsules was pH-dependent, with greater release observed under simulated gastric conditions (pH 1.2) than in simulated intestinal fluid (pH 6.8), in agreement with previous reports [9,28]. Notably, MDGA-coated microcapsules retained significantly higher TPC in the intestinal phase, suggesting enhanced resistance to enzymatic degradation and improved stability under near-neutral pH conditions, which is characteristic of polysaccharide-based encapsulation systems [9,28,33].

The release pattern of MDGA microcapsules in simulated intestinal fluid indicates a controlled-release mechanism closely linked to the physiological conditions of the gastrointestinal tract. Although partial diffusion of phenolic compounds occurred in simulated gastric fluid (pH 1.2), the MDGA matrix exhibited greater resistance to acid penetration, likely due to its compact amorphous structure and reduced molecular mobility. Upon transition to simulated intestinal fluid (pH 6.8), the elevated pH promoted swelling and partial erosion of the maltodextrin–Gum Arabic network, while exposure to intestinal enzymes facilitated matrix relaxation and diffusion of encapsulated phenolics. This combined effect enhanced TPC release in the intestinal phase, demonstrating that MDGA microcapsules effectively protect phenolic compounds from premature gastric degradation and promote their targeted delivery to the intestine, where absorption predominantly occurs. These findings support the role of MDGA as a pH-responsive and enzyme-sensitive carrier that improves the bio accessibility of phenolic compounds.

The release pattern of MDGA microcapsules in SIF further confirms a controlled-release mechanism governed by gastrointestinal conditions. While all samples exhibited partial phenolic diffusion in SGF (pH 1.2), the MDGA matrix showed greater resistance to acid penetration due to its compact amorphous structure and reduced molecular mobility. When transferred to SIF (pH 6.8), increased pH promoted swelling and partial erosion of the maltodextrin–Gum Arabic network, and intestinal enzymes enhanced matrix relaxation and diffusion of encapsulated compounds, resulting in higher TPC release in the intestinal phase. This indicates that MDGA protects phenolics from premature gastric degradation and promotes their targeted delivery to the intestine, where absorption predominantly occurs, thereby improving the bio accessibility of phenolic compounds.

Overall, the digestion behavior of the encapsulated samples was governed by the combined effects of coating material composition, pH environment, and enzymatic activity, which are recognized as key factors influencing phenolic stability and release under simulated gastrointestinal conditions [44,45]. Direct comparison with the non-encapsulated extract therefore clearly demonstrates that MDGA-based microencapsulation improves gastrointestinal stability and controlled intestinal release of *V. sinaiticum* phenolic compounds, supporting their application in functional food and nutraceutical formulations.

### 3.7. Storage Stability Test

**Figure 5:**
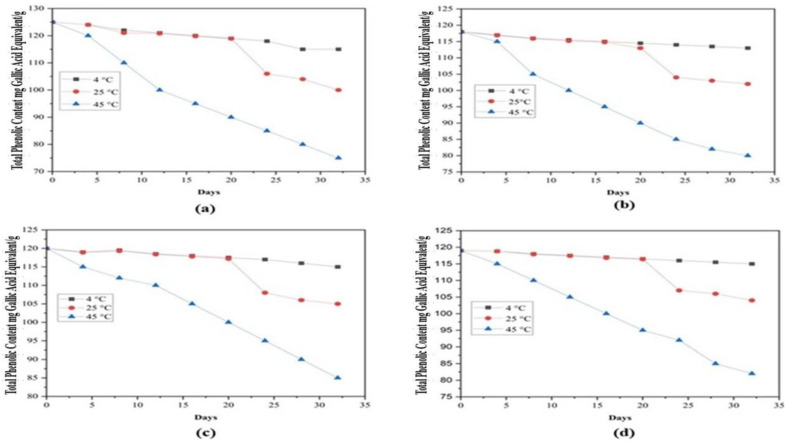
Storage stability based on the Total Phenolic Content (TPC) on coating material: (**a**) TPC; (**b**) GA; (**c**) MD and (**d**) MD-GA: mix, GA: Gum Arabic, MD: maltodextrin, and TPC: total phenolic content.

The storage stability of microcapsules with different coating materials was evaluated over 32 days at three temperatures: 4 °C, 25 °C, and 45 °C, using *V. sinaiticum* extract as a control. During the first 20 days, TPC rapidly decreased at 4 °C (Figure 5), likely due to the loss of surface phenolic compounds in the microcapsules. This aligns with findings by [28,31], who observed a faster decline in TPC during the first 20 days of storage compared to the subsequent 90 days. After 28 days at 45 °C, the *V. sinaiticum* extract showed a 30% loss in TPC, while encapsulated samples (MDGA, MD, and GA) exhibited 29.16%, 31.09%, and 32.20% losses, respectively (Figure 5). After 32 days, statistically significant differences (*p* < 0.05) were observed between MD and GA microcapsules, indicating that coating material influenced long-term phenolic retention.

At 25 °C, the total phenolic content (TPC) of *V. sinaiticum* extract decreased by 16.80% over 28 days, whereas microencapsulation reduced this loss to 11.60% (MD), 12.71% (MDGA), and 12.26% (GA), highlighting the protective role of encapsulation. The most stable formulations were stored at 4 °C, where TPC losses were minimal: 3.36% (MDGA), 4.16% (MD), and 4.23% (GA). These results confirm that lower storage temperatures significantly enhance phenolic stability, with the extract powder itself losing only 5% at 4 °C. Among the coating systems, MDGA consistently provided the greatest protection of phenolic compounds.

Notably, a pronounced decline in TPC was observed between days 20 and 24 for all samples stored at 25 °C (Figure 5). Because oxidative markers, residual enzyme activity, and microbial growth were not directly measured in the present study, the exact mechanism responsible for this sudden decrease cannot be experimentally confirmed. However, similar non-linear degradation patterns of phenolic compounds during ambient-temperature storage have been widely reported and are commonly attributed to temperature-dependent stability transitions in phenolic-rich matrices [46,47,48,49]. A sharp TPC decline between days 20 and 24 across all samples stored at 25 °C suggests the involvement of multiple degradation mechanisms. Phenolic compounds are highly sensitive to environmental factors such as light and pH, in addition to temperature, which may have accelerated degradation during this period. While microencapsulation enhances stability, phenolic compounds remain vulnerable to oxidation and polymerization over prolonged storage. Moreover, minor storage inconsistencies or handling variations during the storage period cannot be completely excluded and may have contributed to the observed sudden decrease in TPC. Importantly, this sharp decline occurred only at 25 °C and was not observed at 4 °C or 45 °C, indicating a temperature-specific stability transition rather than simple heat-driven degradation.

Moderate storage temperatures can increase molecular mobility and cumulative thermal stress, thereby accelerating phenolic degradation through oxidative reactions or enzyme-mediated pathways compared with refrigerated conditions [47,48]. At 25 °C, polyphenol oxidase (PPO) and peroxidase (POD) may remain partially active, catalyzing the conversion of phenolics into quinones that subsequently polymerize into insoluble compounds not detectable by TPC assays [21,46]. In contrast, enzymatic activity is suppressed at 4 °C and largely inactivated at 45 °C, resulting in more gradual and predictable degradation trends.

Polymerization and complexation reactions may further contribute to the sudden loss of detectable phenolics at 25 °C, as phenolic compounds can interact with proteins, polysaccharides, or other macromolecules within the matrix, reducing the concentration of free phenolics [9]. In addition, storage-related factors such as moisture fluctuations and water activity at moderate temperatures may promote hydrolysis or leaching of phenolics, particularly in hygroscopic systems [49]. These effects are less pronounced at 4 °C and differ mechanistically from the heat-driven degradation observed at 45 °C.

Collectively, previous studies have reported significant TPC reductions in plant extracts stored at approximately 25 °C due to oxidation, enzymatic reactions, microbial metabolism, and polymerization processes [47,48,50,51,52,53]. Accordingly, these mechanisms are discussed here as plausible, literature-supported explanations rather than definitive causes of the observed TPC decline. Further studies incorporating PPO and POD activity assays, microbial enumeration, peroxide value analysis, and monitoring of phenolic polymerization products would be required to conclusively elucidate the mechanisms involved [46,50,51,52,53].

Overall, the storage behavior shown in Figure 5 demonstrates that temperature is a critical determinant of phenolic stability and that microencapsulation—particularly using an MDGA matrix—effectively delays phenolic degradation during prolonged storage. These findings emphasize the importance of optimized storage conditions and appropriate wall material selection for extending the shelf life of phenolic-rich products, in agreement with previous reports on microencapsulated natural bioactives [27].

## 4. Conclusions

This study demonstrates that phenolic extracts from *Verbascum sinaiticum* leaves can be successfully stabilized through freeze-drying microencapsulation using maltodextrin (MD), Gum Arabic (GA), and their binary blend (MD:GA = 8:2). The encapsulation performance and physicochemical properties were strongly governed by the wall material composition, confirming the critical role of carrier selection in preserving phenolic-rich extracts. MD-based microcapsules exhibited the most favorable functional characteristics, including high water solubility, low moisture content and hygroscopicity, and the highest encapsulation efficiency. Structural analyses (FTIR, XRD, and SEM) confirmed effective phenolic entrapment without chemical modification, revealing predominantly amorphous and compact matrices that support enhanced stability. The MDGA system provided synergistic benefits, improving matrix integrity, flowability, and controlled hydration behavior relative to single-carrier systems. Storage and in vitro digestion studies demonstrated that microencapsulation significantly improved phenolic retention and gastrointestinal stability compared with the non-encapsulated extract. MD microcapsules showed the highest phenolic preservation during storage, while MDGA microcapsules enabled controlled intestinal release, indicating improved bio accessibility under simulated physiological conditions. Overall, freeze-drying microencapsulation—particularly using MD alone or in combination with GA—offers an effective and scalable strategy to enhance the stability, functionality, and delivery of *V. sinaiticum* phenolic compounds, supporting their application in functional foods and nutraceutical formulations.

## Figures and Tables

**Table 1:** Formulation of microencapsulation mixture.

Coating Material	MD:GA:DW (*w*/*w*/*w*)	Coating Material–Core (*w*/*w*)
100% MD	(10:0:90)	10:1
100% GA	(0:4:96)	10:1
100% (MD + GA)	(8:2:90)	10:1

MD: maltodextrin, GA: Gum Arabic, and mixed (MD + GA), DW: distilled water.

**Table 2:** Physical properties of *V. sinaiticum* encapsulated and nonencapsulated (pure) extract freeze-dried powder.

Physical Properties		Samples		
	Nonencapsulated	MDGA	MD	GA
Hygroscopicity (%)	21.84 ± 0.56 ^d^	10.57± 0.42 ^a^	14.61 ± 0.04 ^b^	13.38 ± 0.31 ^c^
Moisture content (%)	2.84 ± 0.06 ^c^	1.45 ± 0.01 ^a^	1.36 ± 0.04 ^a^	1.66 ± 0.01 ^b^
Water Solubility Index (%)	74.26 ± 0.45 ^d^	90.43 ± 0.20 ^b^	98.48 ± 0.10 ^a^	86.51 ± 0.34 ^c^
Water activity	0.31 ± 0.02 ^b^	0.22 ±0.01 ^a^	0.19 ±0.01 ^a^	0.21 ± 0.01 ^a^
Water Absorption Capacity (g)	0.11 ± 0.01 ^c^	0.16 ± 0.01 ^b^	0.19 ±0.01 ^b^	0.26 ± 0.03 ^a^
Bulk density (g/mL)	0.14 ± 0.02 ^c^	0.35 ± 0.05 ^a^	0.33 ± 0.01 ^a^	0.20 ± 0.01 ^b^
Tapped density (g/mL)	0.16 ± 0.03 ^c^	0.27 ± 0.01 ^b^	0.40±0.10 ^a^	0.18 ± 0.05 ^c^
Hausner ratio	1.46 ± 0.05 ^a^	1.18 ± 0.03 ^b^	1.12 ± 0.02 ^c^	1.39 ± 0.04 ^a^
Carr’s index (%)	31.72 ± 2.45 ^a^	12.32 ± 2.20 ^b^	9.13 ± 0.09 ^c^	28.31 ± 1.68 ^a^
Angle of Repose (°)	39.85 ± 1.60 ^c^	34.10 ± 1.42 ^a^	31.75 ± 1.49 ^b^	34.60 ± 1.51 ^a^

Data are represented as mean values, different superscripts in the same row indicate significant differences (*p* < 0.05) using Tukey’s test. MDGA: Maltodextrin and Gum Arabic coated mixture sample; MD: maltodextrin coated sample; GA: Gum Arabic coating material.

**Table 3:** Microencapsulation efficiency and phenolic compounds of *V. sinaiticum* freeze-dried powder.

Coating Material	Parameter		
	TPC (mg GAE/g)	SPC (mg GAE/g)	MEE (%)
*V. sinaiticum* extract	160.01 ± 0.78 ^A^	-	-
Maltodextrin (MD)	107.00 ± 0.82 ^A^	20.00 ± 0.08 ^B^	81.31 ± 0.65 ^A^
MD + GA	70.03 ± 1.51 ^A^	19.31 ± 0.09 ^B^	72.41 ± 0.16 ^B^
Gum Arabic (GA)	40.07 ± 0.74 ^C^	11.04 ± 0.02 ^C^	65.00 ± 1.25 ^A^

Values are expressed as mean ± standard deviation. Different superscripted uppercase letters within the same column indicate significant differences (*p* < 0.05). MEE: microencapsulation efficiency; TPC: total phenolic content; SPC: surface phenolic content; GAE: gallic acid equivalent.

## Data Availability

The paper contains the original contributions done during the investigation; for further information, contact the corresponding author.

## Supplementary Materials

The following supporting information can be downloaded at: https://www.mdpi.com/article/10.3390/molecules31030471/s1, Table S1: Preliminary screening of MD:GA ratios.
