# Supplementary material for: Microencapsulation of Phenolic Extracts from Verbascum sinaiticum Leaf Using Maltodextrin and Gum Arabic: Physicochemical Properties, Encapsulation Efficiency, and Storage Stability
Source: Molecules. 2026 Jan 29;31(3):471. doi: 10.3390/molecules31030471 (PMC12899088; doi:10.3390/molecules31030471)
Supplement: Supplementary file 1 [file molecules-31-00471-s001.zip › molecules-4088088-supplementary.pdf]

Supplementary Table S1. Preliminary screening of MD:GA ratios

**MD:GA ratio (w/w)**	**Emulsion stability**	**Hygroscopicity**	**Structural integrity (freeze-dried)**	**Powder flowability**	**Preliminary encapsulation performance**	**Overall assessment**
0:10	High	Very high	Poor (collapsed structure)	Poor	Low	Unsuitable
1:9	High	Very high	Poor–moderate	Poor	Low	Unsuitable
2:8	Moderate	High	Moderate	Poor	Moderate	Suboptimal
3:7	Moderate	High	Moderate	Moderate	Moderate	Suboptimal
4:6	Moderate	Moderate–high	Moderate	Moderate	Moderate	Acceptable
7:3	Good	Moderate	Good	Good	Good	Acceptable
8:2	Very good	Moderate	Very good (intact, uniform)	Good	High	Selected
9:1	Moderate	Low	Moderate	Good	Moderate	Suboptimal
10:0	Low–moderate	Low	Poor (fragile matrix)	Good	Low	Unsuitable
0:20	Very high	Extremely high	Very poor	Very poor	Very low	Unsuitable
20:0	Low	Very low	Poor	Good	Very low	Unsuitable

MD: maltodextrin; GA: gum arabic. Preliminary screening was conducted qualitatively and semi-quantitatively based on emulsion behavior, freeze-dried powder appearance, handling properties, and initial encapsulation performance. The MD:GA (8:2, w/w) ratio was selected as the optimal formulation for subsequent experiments.
